# Merit of an Ursodeoxycholic Acid Clinical Trial in COVID-19 Patients

**DOI:** 10.3390/vaccines8020320

**Published:** 2020-06-19

**Authors:** Subbaya Subramanian, Tinen Iles, Sayeed Ikramuddin, Clifford J. Steer

**Affiliations:** 1 Department of Surgery, University of Minnesota, Minneapolis, MN 55455, USA; subree@umn.edu (S.S.); thealy@umn.edu (T.I.); ikram001@umn.edu (S.I.); 2 Departments of Medicine and Genetics, Cell Biology and Development, University of Minnesota, Minneapolis, MN 55455, USA

**Keywords:** Coronavirus, COVID-19, cytokine storm, ursodeoxycholic acid, clinical trial

## Abstract

Corona Virus Disease 2019 (COVID-19) has affected over 8 million people worldwide. We underscore the potential benefits of conducting a randomized open-label unblinded clinical trial to evaluate the role of ursodeoxycholic acid (UDCA) in the treatment of COVID-19. Some COVID-19 patients are characterized with cytokine storm syndrome that can cause severe and irreversible damage to organs leading to multi-organ failure and death. Therefore, it is critical to control both programmed cell death (apoptosis) and the hyper-immune inflammatory response in COVID-19 patients to reduce the rising morbidity and mortality. UDCA is an existing drug with proven safety profiles that can reduce inflammation and prevent cell death. *National Geographic* reported that, “China Promotes Bear Bile as Coronavirus Treatment”. Bear bile is rich in UDCA, comprising up to 40–50% of the total bile acid. UDCA is a logical and attainable replacement for bear bile that is available in pill form and merits clinical trial consideration.

Coronavirus SARS-CoV-2 as the cause of COVID-19 (Corona Virus Disease 2019) has affected over 8 million people worldwide. One of the consensus observations emerging among COVID-19 patients is the systemic inflammation associated with disease severity [1]. There is mounting evidence showing that some COVID-19 patients are presenting with a cytokine storm syndrome similar to secondary hemophagocytic lymphohistiocytosis (sHLH) and is associated with the degree of disease severity [2]. Studies comparing overlapping clinical features and pathogenesis of COVID-19 and sHLH have shown overlapping markers of inflammation [3]. Therefore, treatment with antiviral drugs alone may not be sufficient to combat COVID-19 related cytokine storm syndrome. Several anti-inflammatory agents have been considered to reduce inflammation in COVID-19 patients [4]. Those infected with SARS-CoV-2 show a significant increase in cytokine profiles such as IL-2, IL-17, and TGFα [5,6]. In the early stages of infection, CD4^+^ and CD8^+^ T cells are critical for defense against SARS-CoV-2. CD4^+^ T cells induce virus-specific antibodies by stimulating B cells. On the other hand, CD8^+^ T cells attack and kill the viral infected cells. To support the immune system, proinflammatory cytokines are produced by helper T cells. However, the increasing viral load can induce apoptosis of T cells, leading to an increased immune response in the patient [7]. The overreaction of inflammatory cytokines, or hyper-inflammatory syndrome, can cause severe and irreversible damages to the lungs and other organs leading to cell death and multi-organ system failure. 

Therefore, it is critical to control both apoptosis and the hyper-immune inflammatory response in COVID-19 patients to reduce the rising morbidity and mortality. While reducing hyper-inflammation may be helpful in COVID-19 patients, overall immune suppression using corticosteroids may lead to delayed virus clearance and enhance mucus and impaired antimicrobial peptide secretion [8]. Notably, a recent study has shown that bile acid metabolites regulate T_h_ and T_reg_ cells differentiation [9] and control immunological homeostasis [10]. Bile acids also control inflammation, in part, via inhibition of NLRP3 inflammasomes [11].

Ursodeoxycholic acid (UDCA), a secondary bile acid, is an existing drug with proven safety profiles that can reduce inflammation. The pro-inflammatory cytokines such as TNFα, IL-1, IL-2, and IL-6 are significantly reduced by UDCA [12]. Based on extensive literature and our experience working with UDCA, we recommend conducting a randomized open-label unblinded clinical trial to evaluate the role of UDCA in the treatment of COVID-19. Discovered at the beginning of the 20th century, UDCA was named as such since it was first identified in bear bile and thought to be an isomer of deoxycholic acid. While bear bile is particularly rich in UDCA, comprising up to 40–50% of the total bile acid pool in some animal species during periods of hibernation, it makes up only ~2–3% of the bile acid pool in humans. UDCA has well-established therapeutic properties, in contrast to the relative toxicity of other more hydrophobic bile acids. In the 1970s, synthetically manufactured UDCA entered into Western medicine as a novel drug to treat certain cholestatic liver diseases such as primary biliary cirrhosis in addition to its ability to reduce gallstone formation. UDCA has distinct physicochemical properties compared with the other bile acids and it is now well documented to be both cytoprotective and anti-inflammatory.

We, and others, have shown that UDCA, an endogenous hydrophilic bile acid, is a potent anti-apoptotic agent. It is remarkable in its pleiotropic action on cells to modulate the death receptor pathway, preserve mitochondrial membrane potential, reduce reactive oxygen species, inhibit translocation of pro-apoptotic Bax to the mitochondrial membrane, decrease the release of cytochrome c, and inhibit caspase activation [13]. In addition, it inhibits the E2F-1/p53/Bax pathway, activates nuclear steroid receptors, modulates endoplasmic reticulum stress-mediated pathways via the unfolded protein response, and activates MAPK and PI3K survival pathways. Mitochondrial stabilization results in improved metabolic activity, reduced oxidative stress, and protection against apoptosis, as well as other pathways involved in cell death [14]. We have used it, as well as its taurine conjugate, tauroursodeoxycholic acid, as an agent to treat animal models of neurodegenerative diseases, including Huntington’s, amyotrophic lateral sclerosis, Parkinson’s, and Alzheimer’s disease. UDCA is not only a potent anti-apoptotic agent but significantly upregulates survival pathways. As a therapeutic agent, UDCA is unique in that it is a natural bile acid with no measurable toxicity, crosses the blood–brain barrier, and can be delivered orally to patients. Notably, the South Korean Food and Drug Administration has already approved the use of UDCA for the treatment of amyotrophic lateral sclerosis [15]. In addition, there are numerous UDCA clinical studies worldwide for a variety of human clinical disorders, including retinitis pigmentosa, macular degeneration, and Huntington’s, Parkinson’s, and Alzheimer’s diseases [16,17]. It was recently reported in *National Geographic* that, “China Promotes Bear Bile as Coronavirus Treatment” [18].

In addition to those reporting that it is uniquely cytoprotective and anti-apoptotic as a therapeutic modality, several publications underscore the remarkable anti-inflammatory and immune regulatory properties of UDCA [19,20,21,22]. Recently, many COVID-19 preclinical models have been developed and used in testing with various drugs [23,24,25]. While these models are useful to understand COVID-19 pathobiology and drug testing, it is unclear to what extent these model systems recapitulate the wide spectrum of symptoms and COVID-19 complications observed in humans. Noticeably, there are no preclinical data available for UDCA treatment using COVID-19 models. In addition, there are no recent updates on the use of bear bile in the treatment of coronavirus in China or its efficacy. However, based on the extensive literature of UDCA and its cytoprotective, immune modulatory, and anti-inflammatory properties, we recommend a clinical trial of UDCA in patients with early symptoms as a prophylactic treatment in the onset of COVID-19, in addition to those who are particularly at high risk with obesity, diabetes, chronic lung and cardiac disease, and any immune-compromised state. It is noteworthy that UDCA has no significant side effects, and is nature’s gift to animals, and especially bears, to protect their cell and organ function during long periods of hibernation. UDCA is a remarkable drug, and its potential use in the treatment of COVID-19 is, in part, warranted by the literature, our studies, and the use of bear bile in China for more than 3000 years to treat a long list of inflammatory disorders.
